# A Survey on Patient and Clinician Satisfaction With a Rapidly Implemented Telephone Consultation Service During the COVID-19 Pandemic

**DOI:** 10.7759/cureus.29325

**Published:** 2022-09-19

**Authors:** Ceri McIntosh, Caoimhin O'Higgins, Jerome Philip, Kelvin Mizen

**Affiliations:** 1 Oral and Maxillofacial Surgery, Hull University Teaching Hospitals NHS Trust, Hull, GBR

**Keywords:** questionnaire survey, attitude towards telemedicine, covid 19, remote consultations, telemedicine experience, covid-19 retro

## Abstract

Aim: The novel coronavirus pandemic presented unique challenges to healthcare organisations on an unprecedented scale. Due to the cessation of routine care, the Humberside Oral and Maxillofacial Surgery department implemented a telephone review service to maintain access to care. This survey study was conducted to gain feedback from patients and staff regarding the recently implemented remote telephone consultation service within the Humberside Oral and Maxillofacial Surgery unit.

Methods: A paper questionnaire comprising 16 questions was posted to 199 patients who underwent telephone review. A further questionnaire was sent via SurveyMonkey (Momentive Inc., San Mateo) to the clinicians involved.

Results: From 199 patients, 93 surveys returned were suitable for inclusion; 7 out of 12 staff replied to the survey. The results showed a high level of satisfaction with the service from both groups. There were also suggestions for future additions to the service, including use for new patient consultations, addition of webcam facilities and further streamlining of suitable cases for remote consultation.

Conclusion: Our study demonstrated high patient and staff satisfaction with telephone consultations. It showed that over one third (37%) of patients were able to be discharged via telephone consultation, helping to maintain access, free up clinical resources and reduce the need for face-to-face clinical attendance, which has been vital throughout the coronavirus disease 2019 (COVID-19) pandemic. We now have suggestions for how this service can be implemented in the longer term within our department, including developing clearer guidelines for inclusion in the service and the possible benefit of video consultation.

## Introduction

Severe acute respiratory syndrome coronavirus 2 (SARS-CoV-2) is a novel coronavirus strain that causes coronavirus disease 2019 (COVID-19), a contagious respiratory illness, which has given rise to the COVID-19 pandemic [1]. The first case in the United Kingdom was diagnosed on January 29, 2020 [2]. Since then, there have been over 22 million confirmed cases in the UK as of August 1, 2022 [3]. Governments around the world primarily implemented limitations on travel and social contacts and strict social distancing guidance. This posed a unique and unprecedented challenge to healthcare providers.

In response to the pandemic, Hull University Teaching Hospitals ceased routine clinical activity. With short notice given for this, the Oral and Maxillofacial Surgery department all but stopped non-urgent treatment and consultations overnight [4,5]. In these exceptional circumstances, a telephone consultation service was quickly set up in order to allow some clinical consultations to continue. This was new to our service, as remote consultations were not something previously offered within our department. Therefore, unlike some more established telemedicine clinics, we were not equipped with remote consultation software (such as Attend Anywhere) and the concept was new to the staff and patients [6]. There was initial trepidation among staff about whether the service could act as a functional replacement for face-to-face consultation and how it would be received by patients.

With this in mind, a feedback survey was undertaken of the patients and staff involved in the service. The aims of this were to gain an insight into patient and staff views on telephone consultations, to identify any benefits to the patient of remote consultation and to determine aspects that may need improvement if this service is to continue.

## Materials and methods

A questionnaire titled ‘Maxillofacial Telephone Clinic Survey’ was posted to all patients above 16 years of age who had telephone consultation appointments between April and mid-July 2020. This was a total of 199 patients. Stamped, addressed return envelopes were also included with the survey. Questions were designed to understand a rough demographic of the patients in terms of age and location in relation to the hospital, as well as to know how they felt about having a telephone consultation and if this was something they would be happy to have again in future. The survey comprised 16 questions, with a mixture of multiple-choice Likert-type questions and free text answers, and was approved by the Trust’s audit department. It was distributed to patients of the Oral and Maxillofacial Surgery department at four hospitals across Hull University Teaching Hospitals Trust and Northern Lincolnshire and Goole NHS Foundation Trust, United Kingdom.

Those questionnaires that were returned to the department either fully or partially completed were included in the analysis. The Likert-style question responses were collated and the free text responses were reviewed and in some cases summarised. Additionally, a survey was approved by the audit department to be sent via SurveyMonkey (Momentive Inc., San Mateo) to 12 consultants, associate specialists and specialty doctors who carried out the telephone consultations. Again, the questionnaires used a mixture of Likert-type and free text responses. These sought feedback on the suitability of the cases managed remotely and how clinicians would like to see the service used in the future. These were sent electronically with one follow-up reminder two weeks later to those who had not yet completed the questionnaire.

## Results

**Figure 1:**
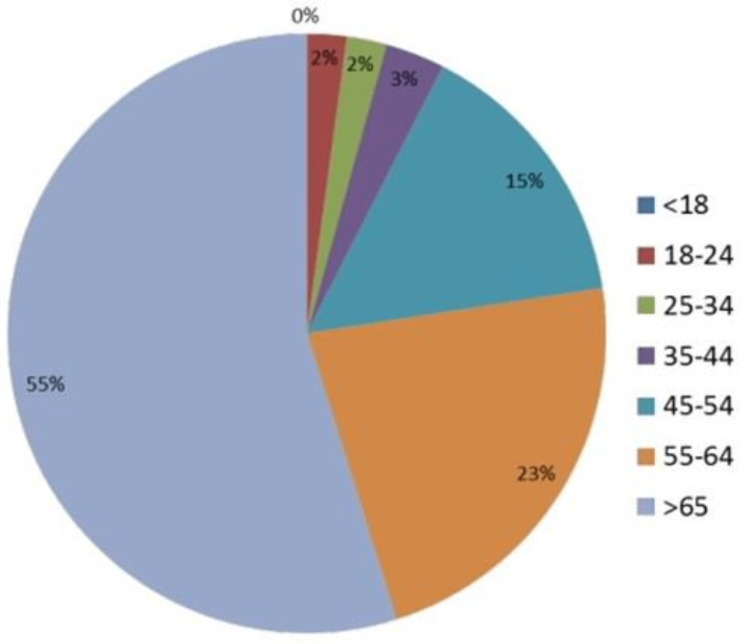
Age distribution of survey respondents

A total of 99 patient questionnaires were returned, and of these, 93 were included for analysis. The six questionnaires excluded from data analysis were returned either blank (two surveys) or with only a few questions answered (four surveys). The over-65s were the largest age group represented, completing 55% of included responses, followed 55- to 64-year-olds who represented a further 23% (Figure 1).

Of 247 total telephone appointments over the period, 37% of patients were discharged from the department by remote consultation; for 53%, further face-to-face appointments at a later date were made. The remaining 10% were either added to treatment waiting lists, were already on an appropriate waiting list or were referred to other specialties or for further investigations. Patient responses are displayed in Tables 1, 2.

**Table 1:** Patient survey responses

Question	Strongly agree	Agree	Neither agree nor disagree	Disagree	Strongly disagree	Other
I was able to fully describe my problems/symptoms over the phone	43	39	4	1	3	3
I felt that I was able to ask questions and have these answered	45	39	4	1	2	1
I felt that the doctor listened to me and understood my concerns	53	32	5	0	2	0
I could understand the doctor clearly over the phone	48	34	4	3	1	1
Were you satisfied over all with the telephone consultation which you received?	47	37	4	1	1	1

**Table 2:** Patient survey responses

Question	Yes	No	Other comments
Were you contacted at approximately the pre-arranged time?	70	8	11
Did you need to take time off work for your telephone consultation?	2	80	
Would you need to take time off work if you came to clinic?	17	74	
Do you have webcam/video calling facilities?	39	52	
Do you think it would be of benefit if you and the doctor could see each other via webcam?	26	49	

Seven members of staff responded to the questionnaire sent out via SurveyMonkey; 5 out of 7 staff members involved in the telemedicine clinics strongly agreed or agreed that they could gather a coherent history over the phone, though they were less confident in reaching a diagnosis. Staff felt that the case mix for the remote clinics was generally appropriate, though in future would like to have more autonomy over which conditions and which of their patients they feel are suitable for telephone assessment. Clinicians could see some benefit to having webcam facilities, which may allow better assessment of certain conditions, e.g. skin lesions; however, they commented that this facility couldn’t replace a face-to-face clinical exam (Tables 3, 4).

**Table 3:** Clinician survey responses

Question	Strongly agree	Agree	Neither agree nor disagree	Disagree	Strongly disagree
I felt that I was able to obtain a clear history over the phone	1	4	1	1	0
I felt confident to reach a diagnosis/advise appropriate management over the phone	0	3	3	1	0
I felt the cases were appropriate for management via telephone	1	3	3	0	0

**Table 4:** Clinician survey responses

Question	Yes	No	Other comments
Would you like to continue with telephone/virtual clinics once routine care recommences?	5	2	
Would webcam facilities widen the scope of patients that you feel would be appropriate for virtual consultation?	3	1	3

## Discussion

The novel coronavirus pandemic presented unique challenges to healthcare organisations on an unprecedented scale. As a result, entire healthcare systems have seen a paradigm shift in how patients are reviewed and treated in hospitals and primary care settings. Strategies such as voice and video calls have been employed to circumvent the challenges posed by limitations on travel and social distancing guidance, to ensure that patients are reviewed in a timely manner.

Telemedicine refers to the remote diagnosis and treatment of patients by using telecommunications technologies [7]. The advantages of telemedicine have been previously documented, including saving time and increasing capacity to provide care [8,9]. Further benefits are the ease of use of telemedicine and convenience for patients as they do not have to leave their home or workplace. Telemedicine has also been associated with more environment-friendly clinical practice, as it reduces the need for travel and also reduces medical waste in the absence of clinical paraphernalia [10]. It has also been shown to allow safe monitoring of long-term conditions and improve access for patients in remote and rural areas [9]. Despite these advantages, there was previously reluctance on the part of clinicians to embrace telemedicine and it was certainly an underutilised modality in our clinical practice. In regard to the COVID-19 pandemic, telemedicine has been particularly useful, as staying at home was mandated by the UK government [11]. This was one of the primary driving forces behind the widespread adoption of telemedicine during 2020 in both primary and secondary care settings. The telephone service allowed triaging of patients to prevent unnecessary journeys to hospital and prioritization of those who did need to be seen. It allowed some element of access to the service to be maintained when routine care was put on hold, improving patient flow through the service and keeping the backlog of cases as low as possible for when routine care resumed.

In primary care, although some GP services could be accessed remotely prior to COVID-19, to many dental practices, this was also a completely new way of working. Maintaining remote triage services was required in order for primary care dental practices to continue receiving NHS funding [12]. The service, which again began almost overnight, was primarily used to deal with urgent cases of pain and infection. However, there is a role for the ongoing remote triage in the COVID-19 recovery phase to help plan patients' treatment prior to attending face-to-face consultations. Subsequently, this allows for a more efficient use of clinical time [13].

This study found that largely, patients were satisfied by the telephone consultations, which is in agreement with previously published studies [14-16]. Interestingly, the majority of patients reported no disruption to work or home life from telephone consultation (80/82 who answered the question) and that clinic attendance would not cause disruption either (74/91 respondents). It is speculated that within the younger age groups, this may have been significantly impacted by the national lockdown at the time of the study, when many were working from home or furloughed. It was not ascertained from this study as to whether telephone clinics would offer an additional advantage to patients when they returned to work, potentially making it easier for them to access care and reduce non-attendance.

The additional dimension added to the consultation by video chat is certainly an aspect to increase the efficiency of an episode of clinical contact. However, concerns regarding the protection of confidential patient information during telemedicine consultations have been identified [17,18]. As such, professional governance bodies and healthcare agencies must work to implement procedures and policies that allow for safe transmissions and discussions relating to patient information.

Within this study, the majority of patients were in the over-65 age group. In the response section of some surveys, it was highlighted that some of these patients may not have access to or be able to effectively use certain modalities of communication, particularly with reference to video calling. However, a number of patients said they did have mobile phones with a camera and were well practiced in using these with apps such as Zoom and FaceTime. The majority of patients surveyed (54%) did not think there would be a benefit to using webcam facilities. Nevertheless, many commented that this would depend on their condition, or whether the doctor had previously examined them in person.

The opinions of clinicians were also canvassed during this study. The addition of video calling to our telemedicine service in Humberside was also supported, particularly for patients who may be hard of hearing and relied on lip reading. While the majority agreed that telephone consultations allowed for a thorough history to be obtained, many did not feel confident to make a diagnosis or treatment plan without a physical examination. It was suggested that telephone consultations may be more appropriate for review patients where clinicians are already familiar with the patient, for example, reviewing patients following minor operations, or those with temporomandibular joint disorders, facial pain or benign oral lesions, such as recurrent aphthous stomatitis. It also acts as a triage system to decide if a patient can be discharged from the service, warrants another telephone review or requires a face-to-face appointment. Clinicians would like to implement clearer guidelines on decisions regarding which of their patients they feel are suitable for remote consultation, which again will help with the smooth flow of patients through the referral and assessment system.

The new patient referral service could benefit from remote consultations; however, radiographs or photographs of diagnostic quality will be required with the referrals. This could allow an initial telephone triage to confirm the history of the presenting complaint and a patient’s medical history. This could allow a one-stop visit to the healthcare facility for extractions or benign soft tissue lesions, reducing the number of attendances to tier 2 and secondary care facilities and freeing up resources to increase access.

Clinicians surveyed in this study supported the continuation of these telephone consultations beyond the pandemic. This is largely in keeping with other such studies where similar opinions of clinicians were described [15,19].

## Conclusions

Prior to remote consultation becoming a necessity, it was seldom used within our department. There was some anxiety and reluctance about how this service would work. However, in this study we have demonstrated both patient and clinician satisfaction with telephone consultations. We sought opinions on the benefits of adding video consultation and identified possible barriers towards this, in terms of patient access to the technology. We saw that over one third (37%) of the patients reviewed via phone were able to be discharged, highlighting the important role remote consultation can have in maintaining access, freeing up clinical resources and reducing costs associated with attendance to clinics. It seems that many in both primary and secondary care are likely to be adopting remote consultation more than ever before. Those in primary care medicine and dentistry have become adept in the skill of remote triage, which is an asset to the continuity of these services now and in any future pandemics. Having introduced this new service to our department, our clinical teams and patients have discovered a different yet effective way of accessing and providing healthcare, which we can now continue and engrain further into our routine clinical practice.
